# In vivo magnetic recording of neuronal action potentials using a differential TMR magnetrode

**DOI:** 10.1038/s41378-026-01421-y

**Published:** 2026-09-04

**Authors:** Yi Wang, Jin Shan, Chenglong Zhang, Zhenhu Jin, Yilin Song, Xinxia Cai, Jiamin Chen

**Affiliations:** 1 State Key Laboratory of Transducer Technology, Aerospace Information Research Institute, Chinese Academy of Sciences, Beijing, 100190 China; 2 School of Electronic, Electrical and Communication Engineering, University of Chinese Academy of Sciences, Beijing, 100049 China; 3 College of Materials Sciences and Opto-Electronic Technology, University of Chinese Academy of Sciences, Beijing, 100049 China

**Keywords:** Biosensors, Biosensors

## Abstract

Neuronal magnetic signal recording intrinsically provides vector information and tissue transparency, offering a potential route to overcome the spatial resolution limitations of conventional electrophysiological recordings. However, in situ detection of neuronal magnetic signals at the cellular level remains highly challenging due to the limited sensitivity of microscale magnetic sensors and the presence of background noise. Here, we report a high-sensitivity implantable differential magnetrode based on a dual-pinned magnetic tunnel junction (TMR). By implementing a spatially decoupled differential architecture with a long baseline (5 mm), together with a sensitivity dynamically matched interface circuit, the device effectively suppresses environmental common-mode noise and achieves an ultralow detection limit of 68 pT/√Hz at 1 kHz. Benefiting from an optimized bidirectional SiO₂/Si₃N₄ protective interface, the device exhibits good biocompatibility and stability. Using this magnetrode, we detected action potential-related magnetic signals in the CA1 region of the rat hippocampus. Crucially, we experimentally observed polarity reversals in spike waveforms that correlate with neuronal spatial orientation, demonstrating the potential of magnetic recording to distinguish neural current directionality based on vector information. This work provides a novel sensing tool for microscale neuroscience research and offers a new technical pathway for resolving the spatial topology of complex neural circuits.

**Figure:**
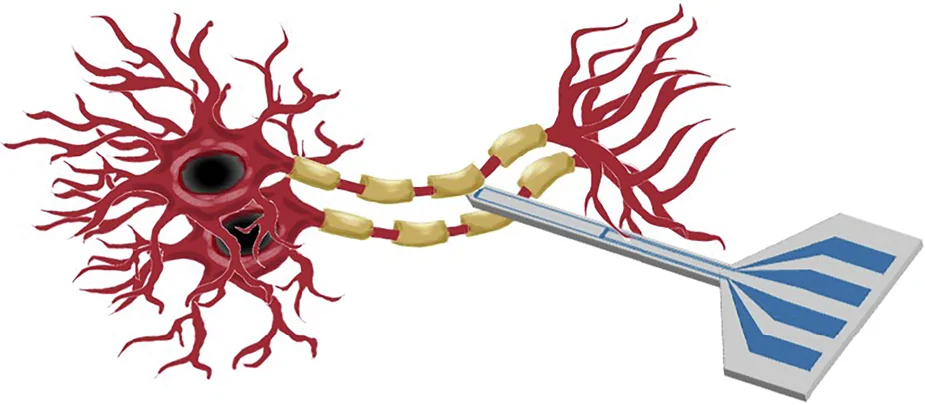

## Introduction

Neuronal action potentials, as the fundamental units of neural information transmission, exhibit spatiotemporal characteristics that directly reflect neuronal activity states, network connectivity topology, and neural encoding mechanisms^1,2^. For decades, electrophysiological techniques have served as the cornerstone of neuroscience research owing to their high temporal resolution and signal-to-noise ratio^3–5^. However, electrical recordings intrinsically measure local potential fluctuations, which are scalar in nature, and their spatial resolution as well as sensitivity to current directionality are constrained by multiple factors, including the inhomogeneous electrical conductivity of biological tissues, the complex electrode–tissue interface impedance, and the configuration of reference electrodes^6,7^. These factors not only limit the reconstruction of the spatial organization of neural currents but also obscure the discrimination of signals originating from different neurons and their processes. Moreover, during chronic implantation, inflammation-induced glial scarring often encapsulates the recording sites, further increasing interface impedance and degrading recording stability, thereby restricting the long-term effectiveness of electrical electrodes^8,9^. In contrast, transient currents generated by neural activity induce corresponding magnetic fields in space. Magnetic signals do not require reference electrodes, are insensitive to tissue conductivity variations due to the uniform magnetic permeability of biological tissues, and inherently possess vector information^10–13^. Consequently, neuronal magnetic signal detection is regarded as an important complement to electrophysiological recording, offering a new perspective for resolving neural circuits and hierarchical structures.

In recent years, various magnetic sensing technologies have been explored for neuronal magnetic signal detection. Among them, superconducting quantum interference devices (SQUIDs)^14–16^ and optically pumped magnetometers^17–19^ (OPMs) exhibit outstanding sensitivity and have demonstrated excellent performance in neural magnetic measurements. However, due to their large physical size, stringent operating conditions, and complex system integration, these techniques are primarily limited to extracranial, macroscale measurements and remain unsuitable for cellular-level neural signal detection. To overcome this spatial resolution bottleneck, spintronic magnetoresistive sensors, characterized by high sensitivity, room-temperature operation, miniaturized dimensions, and excellent compatibility with MEMS fabrication, provide a practical technological pathway for microscale neuronal magnetic detection^20,21^. In 2016, the concept of the “magnetrode” was first proposed^22^, in which highly sensitive, miniaturized magnetoresistive sensors were integrated onto microprobes, analogous to neural electrodes, enabling in situ detection of local neural signals. Subsequent studies predominantly employed giant magnetoresistance (GMR) sensors owing to their relatively simple fabrication processes and technological maturity, achieving successful recordings of population-level hippocampal activity in vitro as well as microscale neural magnetic signals from the visual cortex in vivo^23–26^. Despite demonstrating the feasibility of microscale neural magnetic recording, GMR-based magnetrodes remain constrained by their limited magnetoresistance ratio and intrinsic noise, posing challenges in both sensitivity and signal-to-noise ratio for action potential magnetic signal detection. With continued advances in spintronics, tunneling magnetoresistance (TMR) sensors, offering higher magnetoresistance ratios and sensitivities, have emerged as particularly promising candidates for ultralow magnetic field detection in biomagnetic applications.

In this study, we designed and fabricated a high-sensitivity, low-noise implantable differential TMR magnetrode for in vivo neuronal magnetic signal detection. By incorporating a differential sensing architecture together with customized interface circuitry, environmental common-mode noise was effectively suppressed and the intrinsic device noise was reduced, achieving a magnetic field detection limit of approximately 68 pT/√Hz at 1 kHz, which is superior to previously reported GMR-based magnetrodes. We systematically compared the detection performance of magnetrodes with different reference-unit distributions and verified, through in vitro simulation experiments, the significant signal-to-noise ratio enhancement enabled by the differential architecture. To ensure stability in biological environments, a dual-passivation structure composed of SiO₂/Si₃N₄ layers was introduced. Both in vitro cellular assays and in vivo histological analyses consistently demonstrated that the dual-passivation structure effectively suppresses inflammatory responses and improves device biocompatibility. In in vivo rat experiments, the differential TMR magnetrode recorded action potential-related spike-like magnetic signals. Notably, classification analysis of the recorded spike signals revealed pronounced polarity reversal phenomena. These polarity differences reflect the projection of action potential current directions onto the sensor’s sensitive axis. This result suggests that magnetic recording, owing to its intrinsic directional sensitivity, can distinguish the orientation of neuronal currents relative to the sensor. Consequently, it provides access to directional information on neuronal morphology and signal propagation pathways that is difficult to obtain using conventional electrophysiological techniques.

## Results and discussion

### Design and performance characterization of the differential magnetrode

To achieve high signal-to-noise ratio detection of weak neuronal magnetic signals in deep brain regions, we developed an implantable differential TMR magnetrode based on microelectromechanical systems (MEMS) technology. The magnetrode adopts a unique differential sensing architecture, in which a sensing unit and a reference unit are respectively integrated at the probe tip and at a vertically separated position along the probe axis, forming a spatially differential magnetic sensing structure. This configuration effectively suppresses common-mode noise originating from the environment and readout circuitry. Each magnetic sensing unit consists of 12 magnetic tunnel junctions (MTJs) with dimensions of 15 μm × 45 μm connected in series, which maximizes the device sensitivity while simultaneously reducing 1/f noise^27^.

**Fig. 1:**
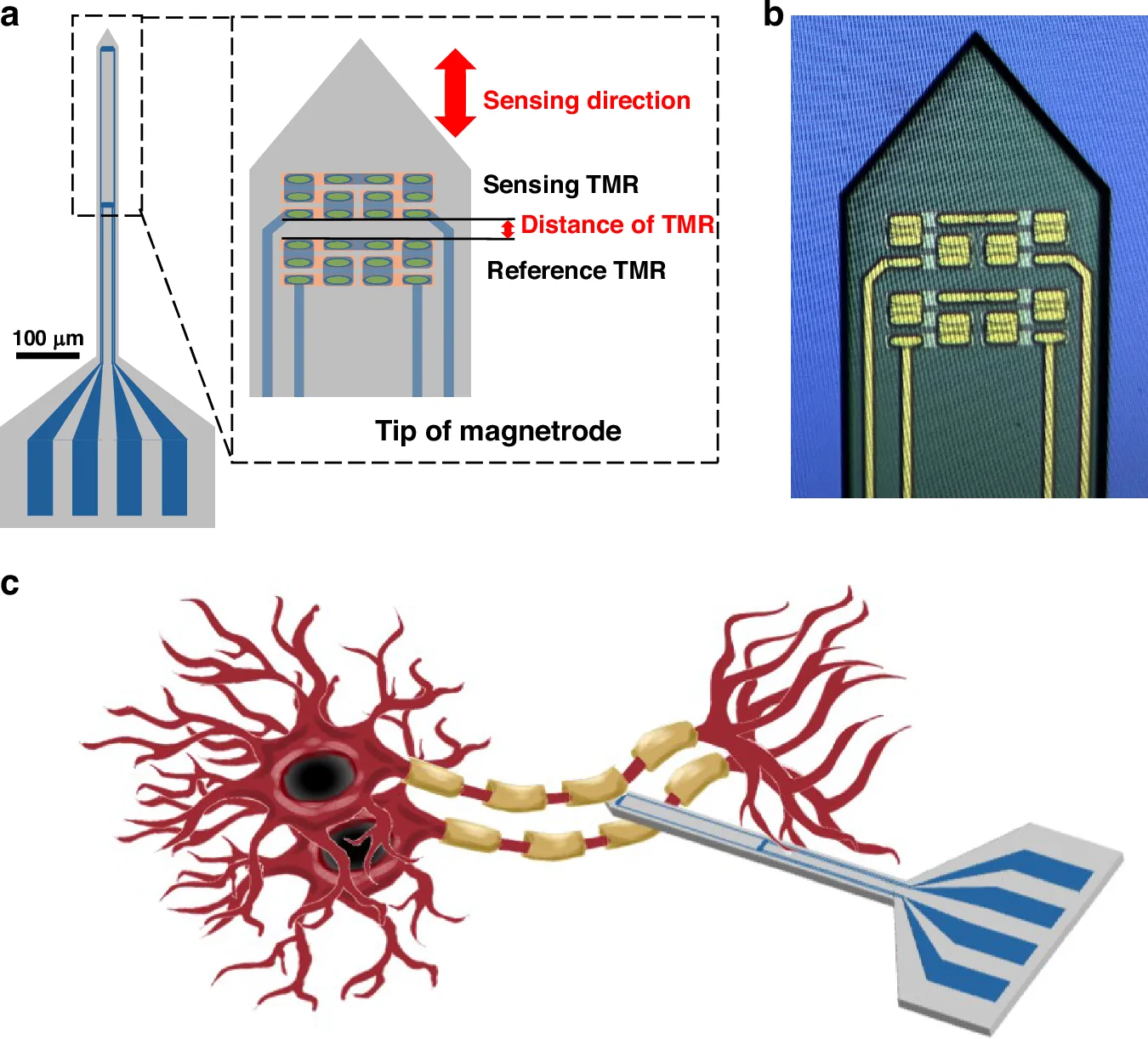
Schematic of the magnetrode to detect magnetic fields generated by action potentials. **a** Schematic of the magnetrode shape and TMR layout. **b** Optical micrograph of the tip of the magnetrode. **c** Schematic of local magnetic field recordings related to neuronal action potentials

Notably, a double-pinned spin-valve structure was employed in the TMR film stack. Compared with conventional single-pinned designs, this configuration improves the consistency of free-layer magnetization reversal, resulting in a stable and symmetric linear response around zero magnetic field. Such a response is essential for high-fidelity neuronal magnetic signal recording. In addition, to mitigate potential mechanical damage to brain tissue during implantation, the silicon substrate thickness was reduced to 200 μm, thereby decreasing the probe cross-sectional area, alleviating implantation-induced tissue injury and inflammatory responses, and improving the overall biocompatibility of the device (Fig. 1).

**Fig. 2:**
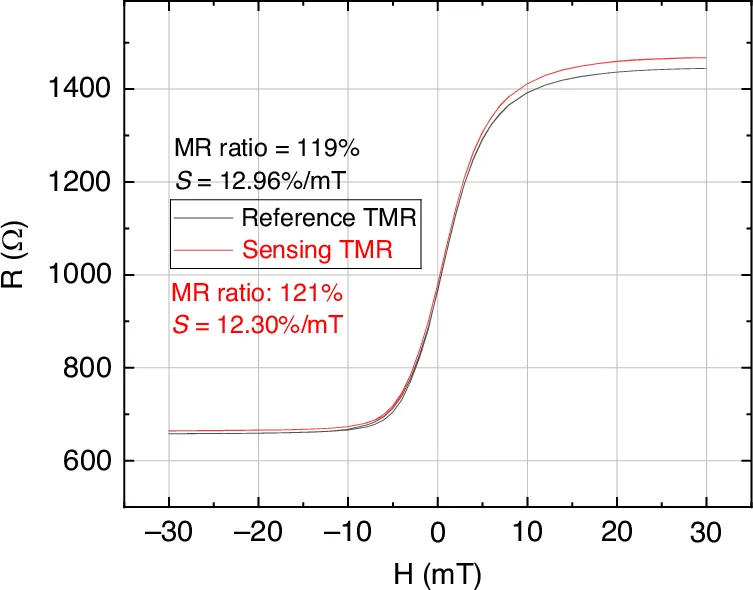
H-R curve of the sensing and reference units in the fabricated TMR magnetrode

Subsequently, the magnetoresistive response characteristics and intrinsic noise performance of the fabricated TMR magnetrode were systematically characterized. Under standard four-probe measurement conditions, both the sensing and reference units exhibited typical and highly consistent TMR responses, with magnetoresistance ratios of 121% and 119%, respectively. The corresponding sensitivities in the linear region reached 12.30%/mT and 12.96%/mT (Fig. 2), demonstrating excellent sensitivity and magnetoresistance performance that satisfy the fundamental requirements for highly sensitive biomagnetic signal detection.

Notably, although the two units exhibit closely matched characteristics, slight discrepancies in resistance and sensitivity remain, which are attributed to inevitable micro- and nanofabrication variations. Such mismatches can degrade the effectiveness of differential measurements if left uncompensated. Therefore, an adaptive differential interface circuit was further introduced to dynamically match the responses of the two channels, as discussed in section “Interface circuit design and signal acquisition”.

**Fig. 3:**
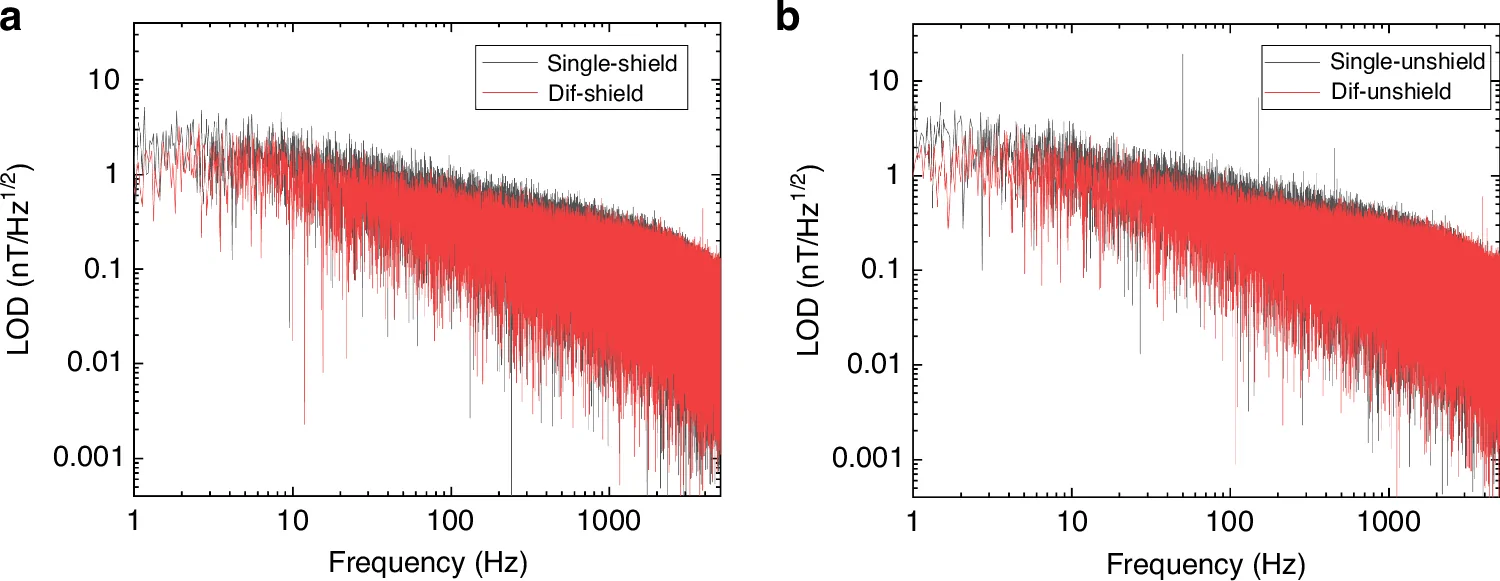
Noise level comparison between single ended and differential TMR magnetrode under varied measurement environments. **a** Noise spectral density measured inside a magnetically shielded chamber. **b** Noise spectral density measured under unshielded ambient laboratory conditions. (LOD limit of detection)

The intrinsic noise characteristics of the device were further evaluated under stringent electromagnetic shielding conditions. During the measurements, the magnetrode was placed inside a magnetic shielding chamber and powered by batteries to further minimize external electromagnetic interference. Figure 3a compares the noise performance of a single-ended TMR configuration and the differential TMR configuration under shielded conditions. Owing to the effective cancellation of background common-mode noise enabled by the differential architecture, the detection limit at 1 Hz was reduced from 1.42 nT/√Hz in the single-ended mode to 1.03 nT/√Hz. More importantly, in the 1 kHz frequency band, where the spectral energy of neuronal action potentials is concentrated, the detection limit was further improved to 68 pT/√Hz, significantly outperforming the 107 pT/√Hz achieved in the single-ended mode. This picotesla-level ultralow detection limit surpasses previously reported GMR-based neural magnetrodes, highlighting the sensitivity advantage of the TMR-based differential magnetrode for microscale neuronal magnetic signal detection (Table 1).

**Table 1:** Comparison of the TMR magnetrode with other currently available implantable magnetrodes

Work	MR Ratio (%)	LOD (nT/√Hz)			
		@1 Hz	@5 Hz	@10 Hz	@1 kHz
Valadeiro et al.^23^	4.0	-	54	~25	~3
Caruso et al.^24^	6.1	~22	~10	7	0.37
Klein et al.^26^	-	~30	~10	~8	1
This Work	121	1.03	0.92	0.63	0.068

“ ~ ” indicates values extracted from graphical data in the cited works, “- ” indicates that the data were not reported in the cited works

Moreover, under unshielded ambient conditions, the differential structure exhibited strong interference suppression capability against external electromagnetic noise. To quantitatively evaluate this, the suppression rate (${\eta}_{50{{\rm{Hz}}}}$) for the fundamental 50 Hz power-line interference is defined as:

$${\eta }_{50{{\rm{Hz}}}}=\left(1-\frac{{A}_{{{\rm{dif}}}}\left(50{{\rm{Hz}}}\right)}{{A}_{{{\rm{single}}}}\left(50{{\rm{Hz}}}\right)}\right)\times 100 \%$$

where ${A}_{{{\rm{dif}}}}\left(50{{\rm{Hz}}}\right)$ and ${A}_{{{\rm{single}}}}\left(50{{\rm{Hz}}}\right)$ represent the noise spectral density peak amplitudes at 50 Hz for the differential and single configurations, respectively. Based on the measured spectra (Fig. 3b), the single-ended noise amplitude at 50 Hz reaches 18.961 nT/√Hz, whereas the differential architecture drastically attenuates this peak to 0.433 nT/√Hz. This calculation demonstrates an effective 50 Hz common-mode suppression rate of ~98%.

### Optimization of reference-unit separation in differential TMR magnetrodes

The transient current distribution generated during neuronal firing can be approximated, under near-field conditions, as an equivalent current dipole magnetic source, whose field strength exhibits a steep spatial decay with increasing distance^10,28^. In a differential TMR magnetrode, the spatial position of the reference magnetic sensing unit not only determines the efficiency of common-mode noise suppression, but also directly affects the detection quality of localized neuronal magnetic signals. If the reference sensor is placed too close to the sensing site, partial self-cancellation of the detected signal becomes unavoidable, thereby degrading the overall detection capability. To quantitatively analyze the influence of the reference TMR position on device performance, three differential TMR magnetrodes with different axial separations between the sensing and reference units were designed and fabricated: a short-distance configuration (28 μm, corresponding to the axial MTJ spacing within a single sensing unit), a medium-distance configuration (2500 μm, located at the middle of the probe shank), and a long-distance configuration (5000 μm, positioned at the rear end of the probe). To accurately evaluate signal quality, the signal-to-noise ratio (SNR) was defined as the ratio between the signal peak-to-peak amplitude and the root-mean-square (RMS) value of the background noise, expressed as

$${\mathrm{SNR}}=10\,{{\mathrm{log}}}_{10}\left(\frac{\overline{P}({f}_{{\mathrm{signal}}})}{\overline{P}({f}_{{\mathrm{noise}}})}\right)$$

where $\overline{P}({f}_{{\mathrm{signal}}})$ denotes the average power spectral density (PSD) within a narrow frequency band centered at the excitation frequency, and $\overline{P}({f}_{{\mathrm{noise}}})$ represents the average background noise PSD integrated over the 0–5 kHz bandwidth, excluding the signal window. In addition, the SNR gain of the differential configuration relative to the single-ended configuration was calculated as ($\Delta {{\rm{SNR}}}={{\rm{SN}}}{{{\rm{R}}}}_{{{\rm{dif}}}}-{{\rm{SN}}}{{{\rm{R}}}}_{{{\rm{single}}}}$).

**Fig. 4:**
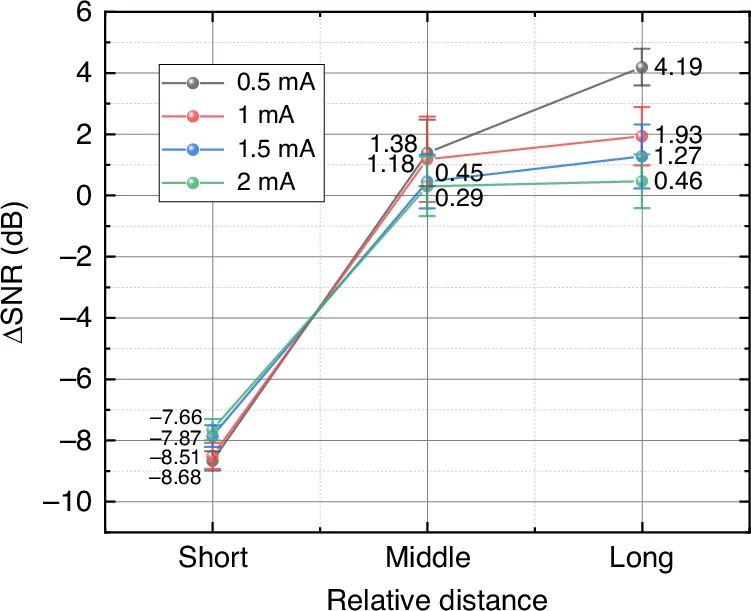
Δ SNR gain of differential TMR magnetrodes with different reference-unit separations, SNR gain of differential TMR magnetrodes measured at four excitation currents (0.5 mA, 1 mA, 1.5 mA, 2 mA). Data points are experimental results; error bars denote standard deviation

Figure 4 summarizes the measured SNR gain for differential TMR magnetrodes with different reference-unit separations. For the short-distance configuration, a pronounced performance degradation was observed: the SNR gain remained negative under all excitation currents, reaching a minimum value of −8.68 dB. This behavior can be attributed to the near-field dipolar nature of neuronal magnetic sources, whose field strength decays rapidly with distance. When the reference and sensing units are positioned within the same decay length scale, both units respond similarly to the localized magnetic signal. As a result, the differential operation suppresses not only common-mode noise but also a substantial portion of the target signal, leading to a reduced SNR and inferior performance compared with the single-ended configuration. As the reference TMR was moved farther away from the sensing site (medium-distance configuration), a moderate improvement in SNR was observed. In contrast, the long-distance differential configuration consistently exhibited the highest SNR enhancement under all tested conditions, with a maximum improvement of 4.19 dB. In this configuration, the reference unit was effectively placed outside the influence region of the localized neuronal magnetic field, enabling successful spatial decoupling between the target signal and background noise. This design preserves the weak neuronal magnetic signals at the probe tip while maintaining efficient suppression of environmental noise. Based on these results, a 5 mm baseline was identified as the optimal geometric parameter that balances near-field signal extraction and far-field noise rejection, providing a solid structural basis for subsequent in vivo experiments.

### Biocompatibility of the magnetrodes

Although TMR sensors exhibit excellent magnetic performance, the ferromagnetic elements contained in their multilayer stacks (such as Co, Ni, and Fe) carry inherent risks of cytotoxicity due to potential ion release. To quantify this risk and validate the necessity of effective isolation for implantation, we compared unpassivated devices with those protected by a high-density SiO_2_/Si_3_N_4_ dual-layer passivation. The effectiveness of this interface engineering strategy was first quantitatively evaluated through in vitro experiments. Three experimental conditions were included in the cytotoxicity assessment: a blank group (without device), a control group (device without SiO_2_/Si_3_N_4_ passivation), and an experimental group (device protected by SiO_2_/Si_3_N_4_ dual-layer passivation).

**Fig. 5:**
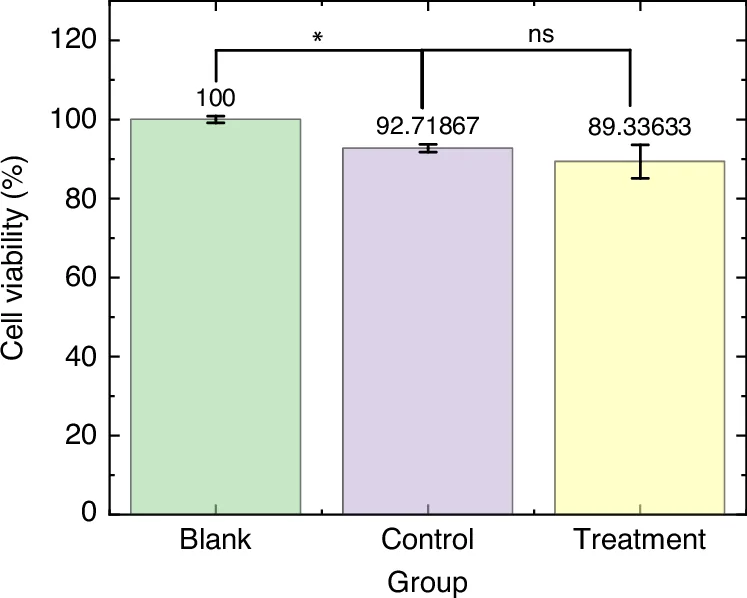
In vitro cytotoxicity evaluation using CCK-8 assay (* indicates *p* < 0.05, and ns indicates no significant difference)

**Fig. 6:**
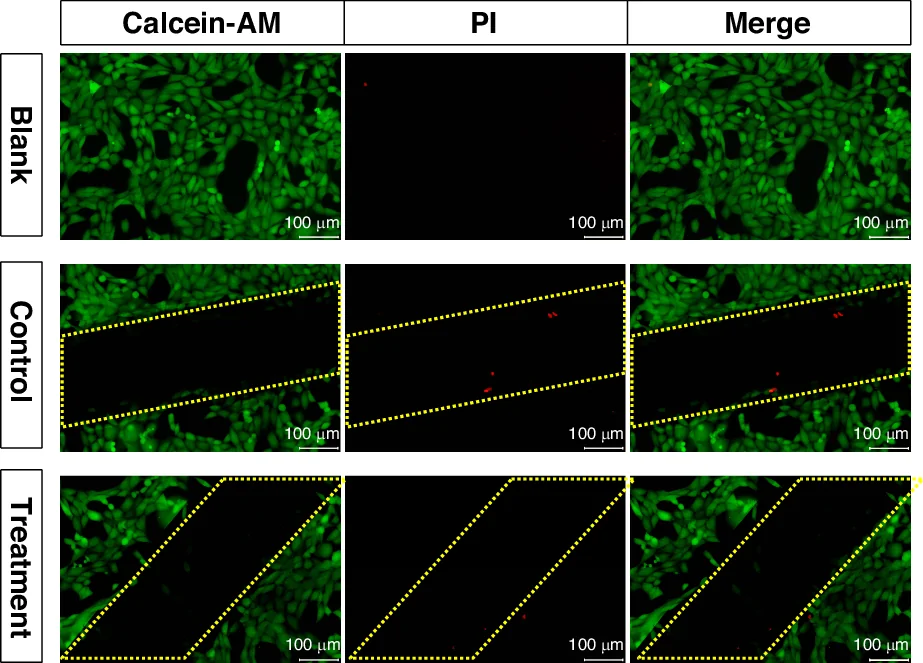
Live/dead fluorescence staining of cells cultured with TMR magnetrode. The central non-fluorescent black rectangular regions, outlined by the yellow dashed boxes, correspond to the geometric silhouettes of the opaque silicon substrates blocking the inverted light paths

The results of the CCK-8 cell viability assay are shown in Fig. 5. With the blank group normalized to 100% (100 ± 0.89%), the relative cell viability in the control group was 89.34 ± 4.22%, while the experimental group was 92.72 ± 0.94%. Statistical analysis using one-way ANOVA revealed a significant decrease in cell viability for the experimental group compared to the blank control (*p* = 0.013 < 0.05). Although the difference between the passivated and unpassivated groups was not statistically significant (*p* = 0.247 > 0.05). Notably, the cell viability of both groups remained far above the threshold for non-cytotoxicity required by relevant international biocompatibility standards. Live/dead cell staining using Calcein-AM/PI further provided intuitive evidence of cellular status. As shown in Fig. 6, the blank group exhibited a high density of green fluorescence (live cells), indicating favorable cell growth. In the control group, scattered PI-positive signals (red fluorescence) were observed near the regions in direct contact with the device, suggesting mild localized damage associated with the exposed sensor surface. By comparison, the experimental group showed markedly fewer red fluorescent signals, with cells displaying well-spread morphologies and densities comparable to those of the blank group. These morphological observations are consistent with the CCK-8 results, confirming that the dual-layer passivation effectively isolates potentially harmful interfacial interactions.

**Fig. 7:**
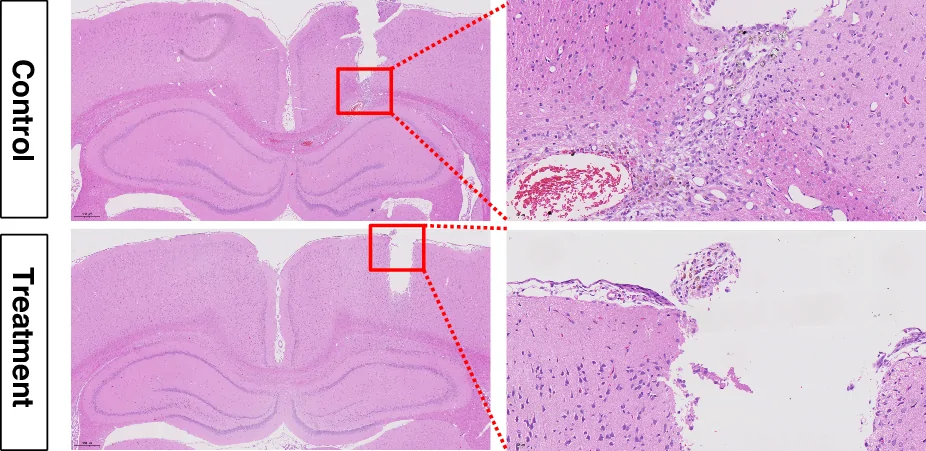
Histological evaluation of in vivo biocompatibility of TMR magnetrode

To further evaluate in vivo tissue compatibility, devices from the experimental and control groups were implanted into the CA1 region of the rat hippocampus (Fig. 7). Hematoxylin and eosin (HE) staining of brain sections at 7 days post-implantation revealed pronounced tissue disorganization and inflammatory cell infiltration around the implantation track in the control group, consistent with the inflammatory response triggered by exposed metallic elements. In contrast, the surrounding brain tissue in the experimental group remained relatively intact, with substantially reduced inflammatory infiltration.

**Fig. 8:**
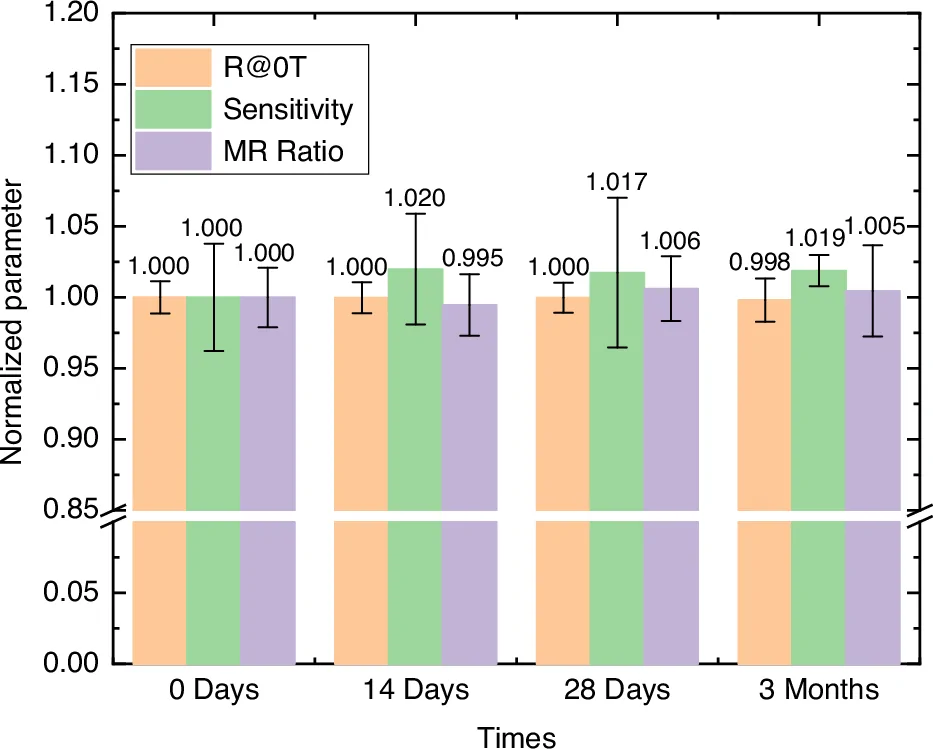
Stability of TMR magnetrode during in vitro aCSF immersion

Taken together, the combined in vitro assays and 7-day in vivo histological assessments demonstrate that the SiO₂/Si₃N₄ dual-layer passivation effectively suppresses acute nonspecific interfacial interactions and inflammatory responses. However, maintaining true biocompatibility for chronic neural interfaces is primarily challenged by continuous exposure to corrosive physiological fluids. Over time, biofluids can cause gradual chemical degradation of bare spintronic thin films, potentially leading to chronic leaching of toxic ferromagnetic ions and triggering severe delayed tissue encapsulation. Therefore, the core value of the dual-layer passivation extends beyond mitigating acute inflammation; it serves as a critical chronic barrier. To verify the durability of the dual-passivation layer structure, we immersed the device in artificial cerebrospinal fluid at 37 °C and conducted 3-month in vitro performance monitoring (Fig. 8). The passivated magnetrode maintained highly stable sensitivity, MR ratio, and zero field resistance, indicating that the presence of the passivation layer effectively prevents fluid infiltration and device structure corrosion. While the current 7-day in vivo H&E staining demonstrates favorable acute tissue compatibility, we acknowledge that this short-term evaluation is insufficient to fully characterize chronic biological responses. Future studies will extend the histological evaluation to longer time points and incorporate GFAP and Iba-1 to quantify astrocytic and microglial reactions, along with utilizing flexible polymer substrates or anti-inflammatory coatings to further optimize the chronic neural interface^29,30^.

### Spike recording and signal processing

To validate the capability of the optimized differential TMR magnetic probe to detect neural activity-related magnetic signals in a complex biological environment, the device was implanted into the CA1 region of the rat hippocampus for in vivo recording. The CA1 region was selected because of its relatively ordered laminar cytoarchitecture and the comparatively consistent orientation of pyramidal neurons^31^. The raw voltage traces were first converted into magnetic-field units according to the calibration coefficient of the device. The calibrated signals were then band-pass filtered between 300 and 3000 Hz using a fourth-order Butterworth filter to suppress low-frequency drift and high-frequency background noise while preserving frequency components associated with spike-like transient events.

Candidate spike events were identified using a threshold-based detection procedure. The detection threshold was set to three times the root-mean-square value of the background noise. A 1-ms refractory window was then applied^32^, and large-amplitude noise artifacts were excluded. For each detected peak, a 3-ms waveform segment centered on the peak was extracted for subsequent morphological analysis. To reduce amplitude-dominated bias during feature extraction and to emphasize waveform dynamics, each extracted waveform segment was normalized by its maximum absolute amplitude. Principal component analysis (PCA) was then performed on the normalized waveforms for dimensionality reduction. The first six principal components, which captured the majority of waveform variance, were selected as input features for unsupervised clustering using the k-means algorithm^33^. For each isolated cluster, the mean waveform and standard deviation, total event number, peak amplitude, signal-to-noise ratio, and inter-event interval distribution were calculated.

**Fig. 9:**
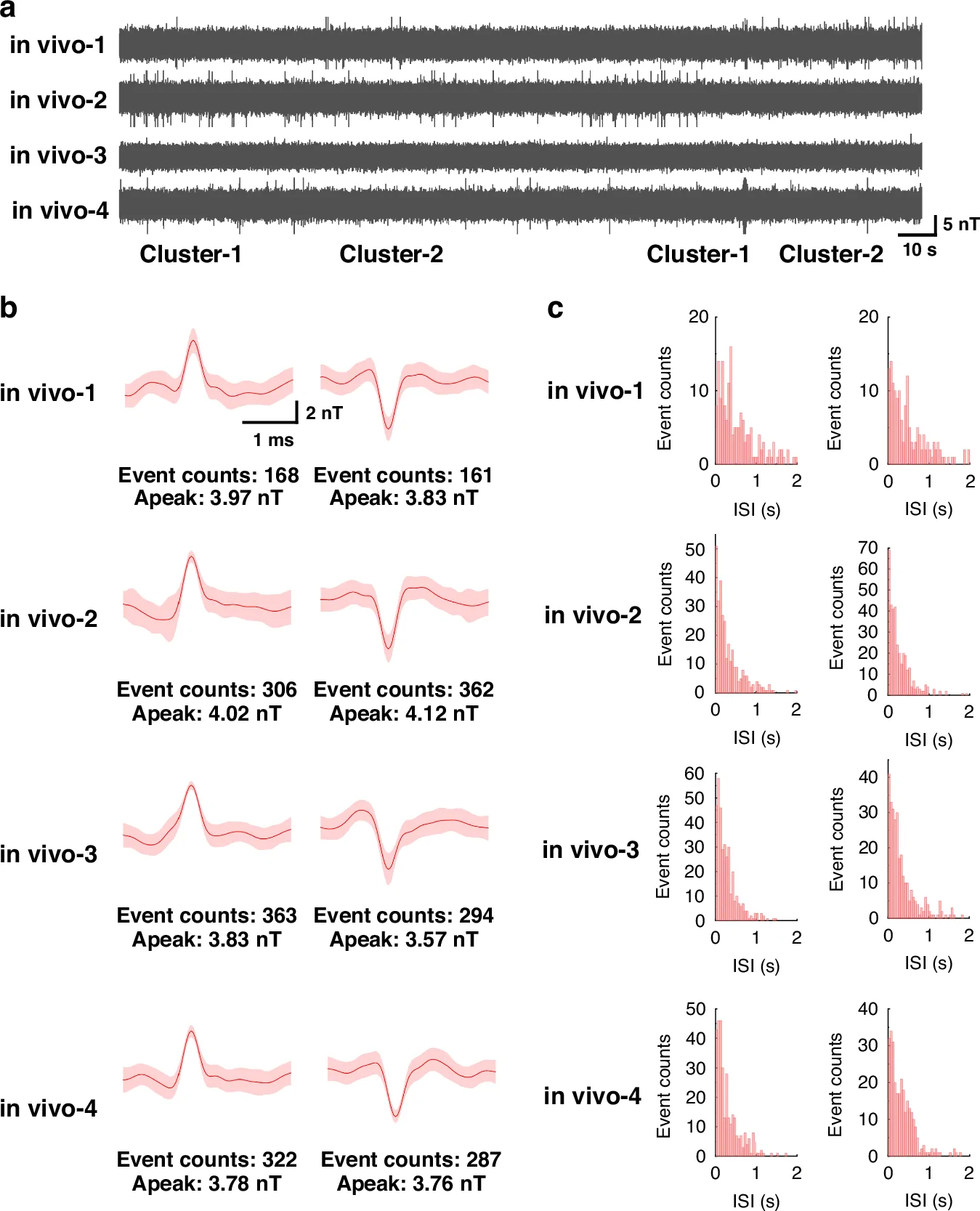
In vivo detection of spike-like magnetic waveforms. **a** In vivo magnetic-field traces recorded from four independent recording trials. **b** Mean waveforms ± standard deviation of two representative waveform clusters. **c** Inter-spike interval distributions of the corresponding waveform clusters

Figure 9 presents four representative sets of in vivo recordings. Reproducible spike-like magnetic waveform clusters were detected in all four recording sessions. In each dataset, two representative waveform clusters with opposite main-peak polarities were observed. Both clusters exhibited clear transient characteristics, with a dominant peak duration of ~1 ms and an overall waveform width of about 2–3 ms. These temporal features are highly consistent with the action potential kinetics observed in the mammalian central nervous system, supporting their possible association with local neural activity of the recorded signals^34,35^. The event rates of these representative clusters were ~1.61–3.63 Hz within the 100-s recording windows, with peak amplitudes of 3.57–4.12 nT and signal-to-noise ratios of 11.05–12.30 dB. Although theoretical biophysical models estimate the magnetic field generated by a solitary unmyelinated axon to be on the order of a few picoteslas, the macroscopic nanotesla-level amplitudes observed in the present study may be physically plausible given the specific cytoarchitecture of the recording target and the geometric footprint of the sensor. The stratum pyramidale of the rat CA1 region is characterized by densely packed pyramidal neurons with a highly parallel arrangement. During semi-synchronous local population firing, the aligned neuronal current components may promote constructive spatial summation of the associated magnetic fields. In addition, the finite physical footprint of the 12-MTJ series sensor allows it to function as a spatial integrator over a local sensing area, thereby capturing the superimposed magnetic flux arising from localized multi-unit activity rather than the magnetic field generated by a single isolated axon. Comparable amplitude ranges have also been reported in previous state-of-the-art magnetoresistive neuro-magnetic recordings from neural tissues.

Notably, the two representative waveform clusters recorded in vivo exhibited opposite main-peak polarities, with one cluster dominated by a positive peak and the other by a negative peak. This polarity reversal demonstrates the unique directional sensitivity of magnetic recordings compared with conventional electrophysiological recordings. Conventional extracellular electrical recordings measure local potential changes relative to a reference electrode, and the observed signal polarity is strongly influenced by the position of the reference electrode, tissue conductivity distribution, and electrode–tissue interface conditions. In contrast, the magnetic field generated by neural currents is intrinsically directional, with its orientation determined by both the direction of current flow and the spatial relationship between the current source and the sensor. According to the Biot–Savart law, transmembrane currents or local current sources propagating along neuronal processes generate circumferential magnetic fields in the surrounding space. When current components with different orientations, or current sources located at different spatial positions, project oppositely onto the sensitive axis of the TMR sensor, magnetic signals with opposite polarities may be recorded. These findings suggest that TMR-based magnetic recording is capable not only of detecting transient magnetic signals associated with local neural activity, but also of providing direction information of local neural currents. In this sense, magnetic-signal polarity may serve as a direction-sensitive feature, offering complementary information that is difficult to obtain from conventional electrophysiological recordings. This feature may be particularly useful in brain regions with laminar structures and relatively consistent neuronal orientations, such as the hippocampal CA1 region, where it may help reveal the spatial organization of local neural activity.

Nevertheless, several limitations should be acknowledged. First, each sensing unit consists of 12 MTJs connected in series and therefore has a finite sensing area. Consequently, the measured output represents a spatial integration of the local magnetic field within the sensing region rather than the response of an ideal point sensor to a single neuron. Second, the current device is a single-axis TMR magnetic probe and can only detect the projection of the local magnetic field along the sensitive axis of the sensor; it cannot reconstruct the full three-dimensional magnetic-field vector. Therefore, the polarity reversal observed in this study should be interpreted as different projections of neural current-related magnetic fields along the sensitive axis of the sensor, rather than as a unique determination of the direction of a single neuron or individual axon. In the future, extending the present single-channel TMR magnetic probe into a high-density, multi-channel array, together with multi-axis magnetic sensing structures or spatial inverse-reconstruction algorithms, may improve spatial resolution, enable discrimination of magnetic-field distributions generated by adjacent neurons or neuronal processes with different orientations, and provide a technical basis for three-dimensional reconstruction of local neural current directions.

**Fig. 10:**
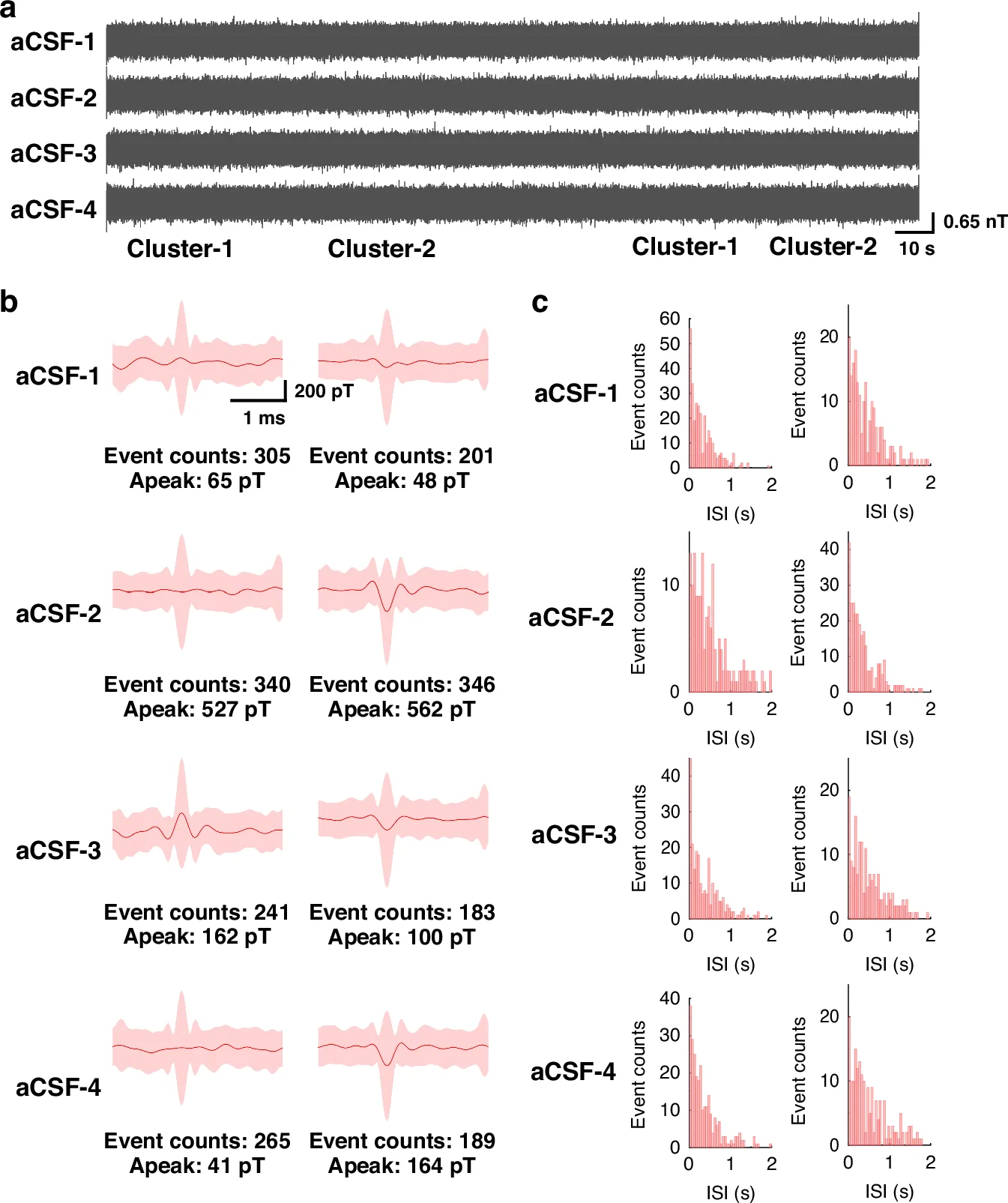
Cell-free aCSF control for assessing non-neuronal magnetic transients. **a** Magnetic field traces recorded in cell-free aCSF across four independent trials. **b** Mean waveforms ± standard deviation for two representative waveform clusters. **c** Inter-event interval distributions of the corresponding magnetic transient clusters

To further evaluate whether these transient magnetic signals could originate from environmental electromagnetic interference, readout circuit noise, or algorithm induced false detections, negative-control experiments were performed in cell-free artificial cerebrospinal fluid. The aCSF control contained no neural tissue or neural discharge source, while the recording system, shielding conditions, sampling rate, amplification parameters, filtering range, and signal-analysis pipeline were kept identical to those used in the in vivo experiments. Figure 10 presents four representative sets of aCSF control recordings. Compared with the in vivo recordings, the aCSF controls mainly exhibited low-amplitude background fluctuations under the same 300–3000 Hz filtering conditions. Although the same detection algorithm could still identify low-amplitude candidate transient events, their peak amplitudes were predominantly within the range of tens to hundreds of picoteslas. The waveform morphology and polarity stability of these candidate events were substantially weaker across different recording segments than those observed in vivo. Moreover, the top principal components accounted for ~83% of the total waveform variance for in vivo recordings, compared with only around 60% for aCSF control data. Combined with the lower and less stable cross-segment correlations of aCSF candidate waveforms, these findings indicate that the in vivo detected neural events exhibit highly stable and structured waveform characteristics. These results indicate that the candidate events detected in aCSF are more likely attributable to residual background fluctuations, readout noise, or occasional transient artifacts rather than stable neural-like magnetic signals.

**Fig. 11:**
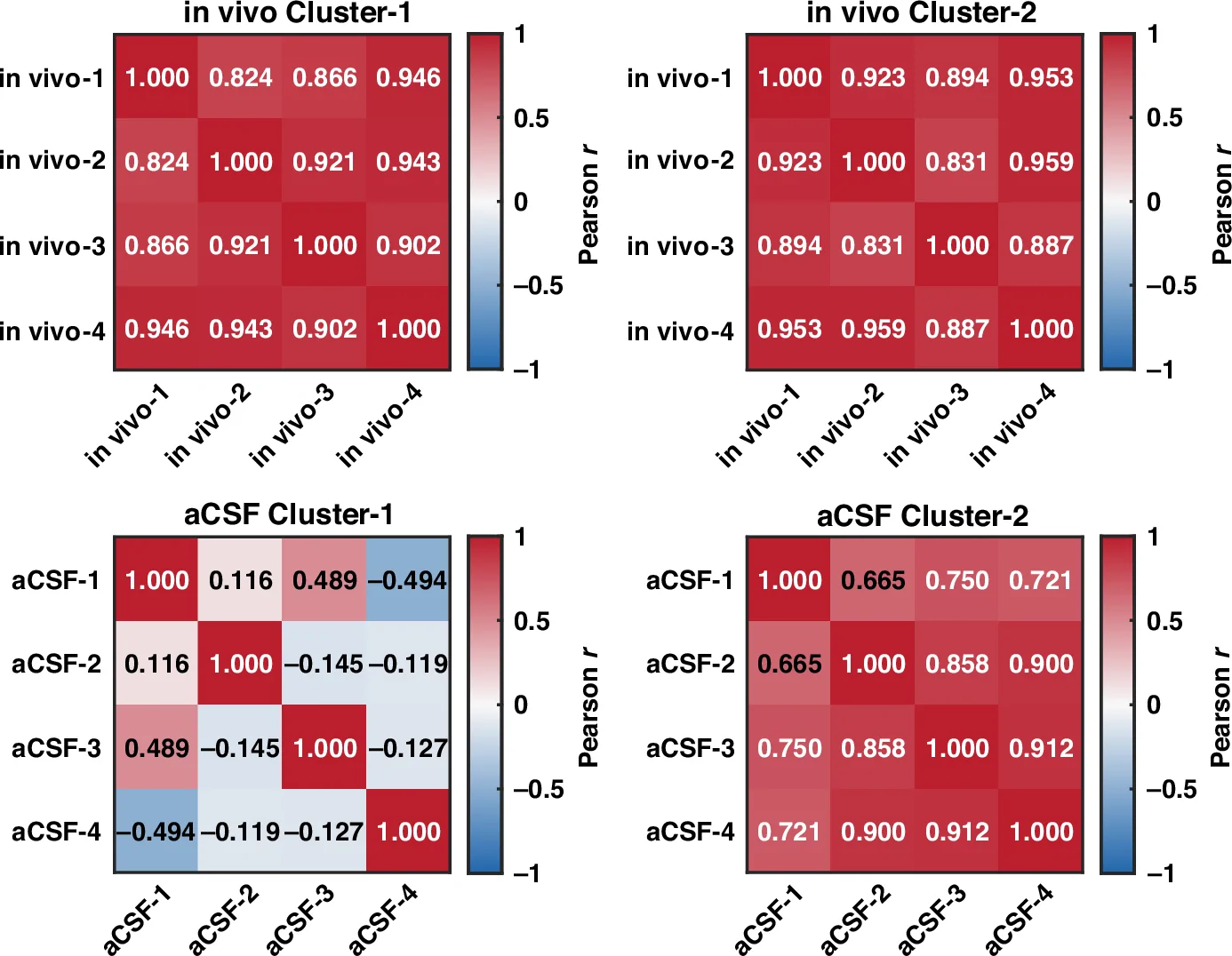
Pearson correlation analysis of same-polarity waveform clusters in in vivo and aCSF recordings

To further compare the stability of waveform structures between the in vivo recordings and aCSF controls, Pearson correlation coefficients were calculated for waveforms with the same polarity across different recording segments (Fig. 11). Pearson correlation coefficient r ranges from −1 to 1, where r close to 1 indicates a strong positive correlation, r close to −1 indicates a strong negative correlation, and r close to 0 indicates weak or no linear correlation. In the in vivo recordings, waveforms with the same polarity exhibited relatively high correlations across recording segments, indicating good cross-segment consistency of the spike-like waveform clusters. In contrast, the candidate waveforms detected in the aCSF controls showed markedly lower correlations, with some same-polarity candidate clusters exhibiting only weak or unstable correlations. These findings further support that the high-amplitude, opposite-polarity, and reproducible waveform structures observed in vivo were not simply caused by random noise or clustering artifacts.

## Conclusion

In this work, we demonstrated a high-performance implantable differential TMR magnetrode capable of recording neuronal action potential–related magnetic signals from the CA1 region of the rat hippocampus at the microscale. To address the intrinsically weak nature of neuronal magnetic fields and their rapid decay in the near-field regime, a differential sensing architecture with a long baseline of 5 mm was established. This spatial decoupling strategy effectively suppressed environmental common-mode noise (power-line interference suppression >98%) while preserving localized weak signals, resulting in an ultralow magnetic field detection limit of 68  pT/√Hz at 1 kHz. This performance represents a clear improvement over previously reported GMR-based neural magnetrodes. Furthermore, a dual-layer SiO₂/Si₃N₄ passivation scheme was introduced, which not only mitigated potential ion release from ferromagnetic materials and improved biocompatibility, but also protected the device from corrosion and degradation induced by complex physiological environments, such as fluid infiltration and protein adsorption. Together, these features supported acute in vivo tissue compatibility and in vitro device stability. In vivo experiments revealed a distinctive spike polarity inversion phenomenon consistent with different projections of local neural current-related magnetic fields. This observation suggests that TMR-based magnetrodes can preserve directional information of neuronal currents at the microscale. By providing this direction-sensitive information, magnetic recording offers a new technical pathway for resolving the spatial topology of complex neural circuits.

Nevertheless, the current single-channel magnetrode still has limitations in fully decoupling neuronal current direction from spatial location. In particular, the use of 12 series-connected MTJs in each sensing unit, while beneficial for increasing the output signal amplitude and improving the equivalent noise performance, inevitably enlarges the effective sensing area. This introduces a trade-off between sensitivity and spatial resolution. As a result, the present sensing unit should be regarded as a finite-area spatial integration detector rather than an ideal point sensor. When magnetic fields generated by neighboring neurons or axonal segments with different orientations overlap within the sensing region, the recorded signal may contain spatially averaged or superimposed components, and opposite-polarity field components may be partially canceled. This effect may limit definitive single-neuron signal separation and precise source localization.

These limitations also highlight the need for high-density arrayed TMR magnetrodes. Owing to the MEMS-compatible fabrication process, the proposed device platform provides a promising basis for array integration. However, the transition from a single-channel magnetrode to a high-density array still involves several technical challenges. Miniaturizing the MTJ elements or reducing the number of series-connected junctions can improve spatial resolution, but may also reduce the output signal amplitude and degrade low-frequency noise performance. Therefore, further optimization of the TMR film stack, junction geometry, and series/parallel configuration will be required. In addition, array integration will increase the complexity of interconnect routing, probe cross-sectional design, packaging reliability, and channel-to-channel calibration. Low-noise, low-power multi-channel readout circuits, together with source-separation and spatial reconstruction algorithms, will also be necessary to reduce crosstalk, thermal load, and external interference, and to distinguish magnetic fields generated by adjacent or differently oriented neuronal current sources. Future work will therefore focus on developing miniaturized, high-density TMR magnetrode arrays with independent multi-channel readout and on-chip signal processing, aiming to improve spatial resolution while maintaining sufficient sensitivity for three-dimensional reconstruction and precise localization of neuronal firing currents.

## Method

### Fabrication of differential TMR magnetrodes

**Fig. 12:**
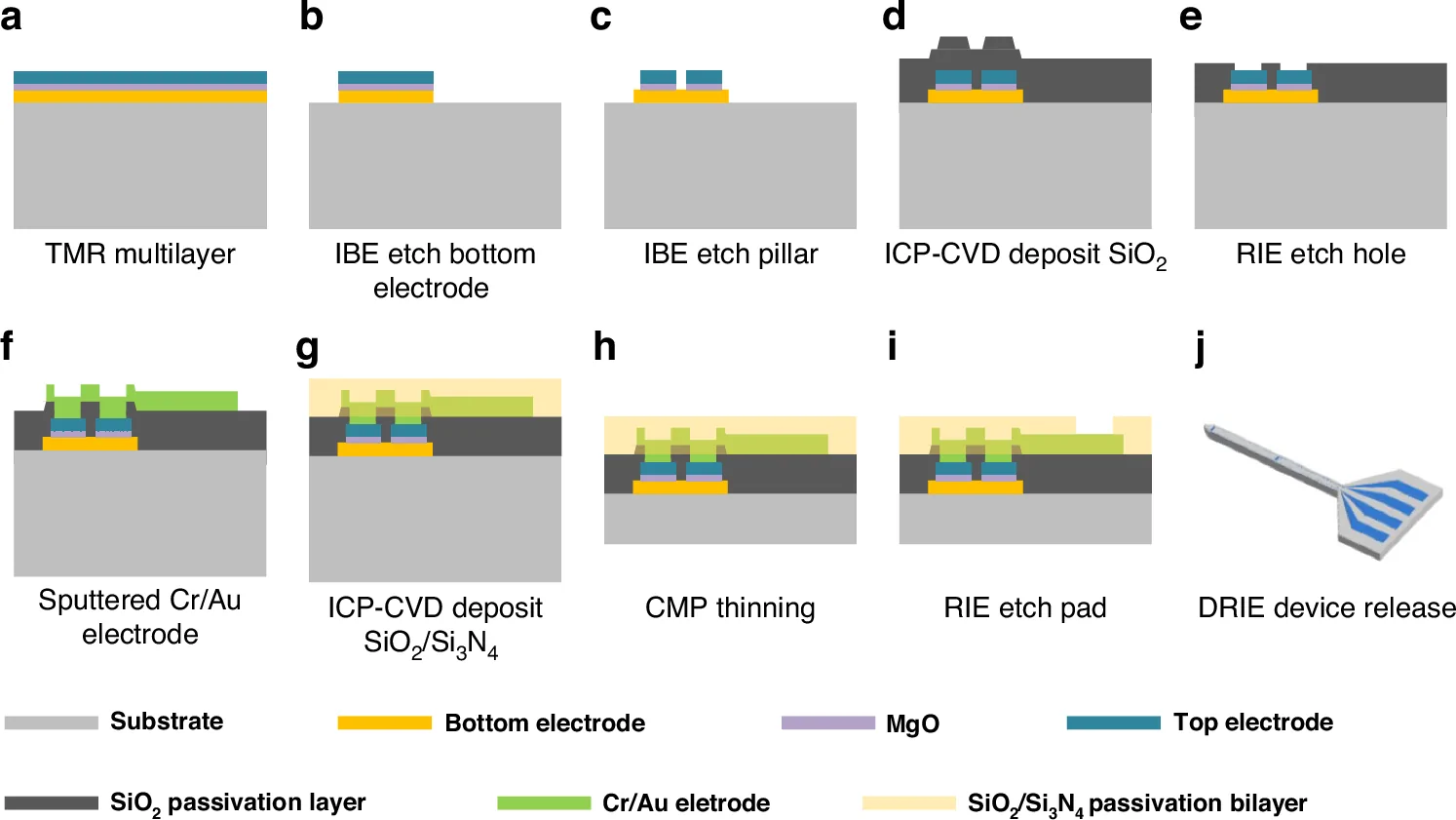
Microfabrication process flow of the implantable differential TMR magnetrode

Device fabrication was performed on wafers pre-deposited with magnetic tunnel junction (MTJ) multilayers featuring a dual-pinned-layer structure. The TMR multilayer stack consisted of: Si/SiO₂/Ta/Ru/Ta/Ru/Ta/Ru/PtMn/CoFe/Ru/CoFeB/ MgO/CoFeB/NiFe/IrMn/Ru/Ta/Ru. Device fabrication was carried out using microelectromechanical systems (MEMS) techniques and consisted of the following key steps (Fig 12).

- MTJ patterning: High-precision MTJ patterning was achieved using a two-step ion beam etching (IBE) process. The first IBE step defined the MTJ junction area, while the second step was used to selectively pattern the free-layer region. To ensure accurate etch termination at the MgO tunnel barrier, real-time endpoint detection was performed using an optical spectrometer^36^. This step is critical for preserving barrier integrity and ensuring consistent magnetoresistive performance (Figure 12a–c).
- Insulation and via opening: A 200 nm-thick SiO₂ insulating layer was deposited by inductively coupled plasma chemical vapor deposition (ICP-CVD) to prevent oxidation and electrical shorting at the MTJ sidewalls. Subsequently, vias were precisely opened in the insulating layer by reactive ion etching (RIE) to enable electrical interconnection (Figure 12d–e).
- Electrode formation and series connection: Cr (30 nm)/Au (200 nm) electrodes were fabricated using a lift-off process. This metallization layer enabled the series connection of 12 MTJs to form a single high-sensitivity magnetic sensing unit, while contact pads were defined for subsequent integration with interface circuitry (Figure 12f).
- Dual-layer passivation: After MTJ fabrication, a dual-layer passivation stack consisting of SiO₂ (200 nm) and Si₃N₄ (200 nm) was deposited by plasma-enhanced chemical vapor deposition (PECVD). This passivation layer served a dual purpose: protecting the MTJ junctions from mechanical damage during subsequent chemical mechanical polishing (CMP), and acting as a biocompatible barrier to reduce nonspecific interactions between the implanted device and surrounding brain tissue (Figure 12g).
- Substrate thinning and shaping: The device surface was protected with photoresist, and the silicon substrate was thinned from an initial thickness of 700 to 200 μm using CMP to reduce tissue invasiveness during implantation. Finally, RIE was employed to expose the contact pads, and deep reactive ion etching (DRIE) was used to define the needle-shaped geometry of the magnetrode (5 mm in length and 350 μm in width), completing device release (Figure 12h–j).

### Interface circuit design and signal acquisition

**Fig. 13:**
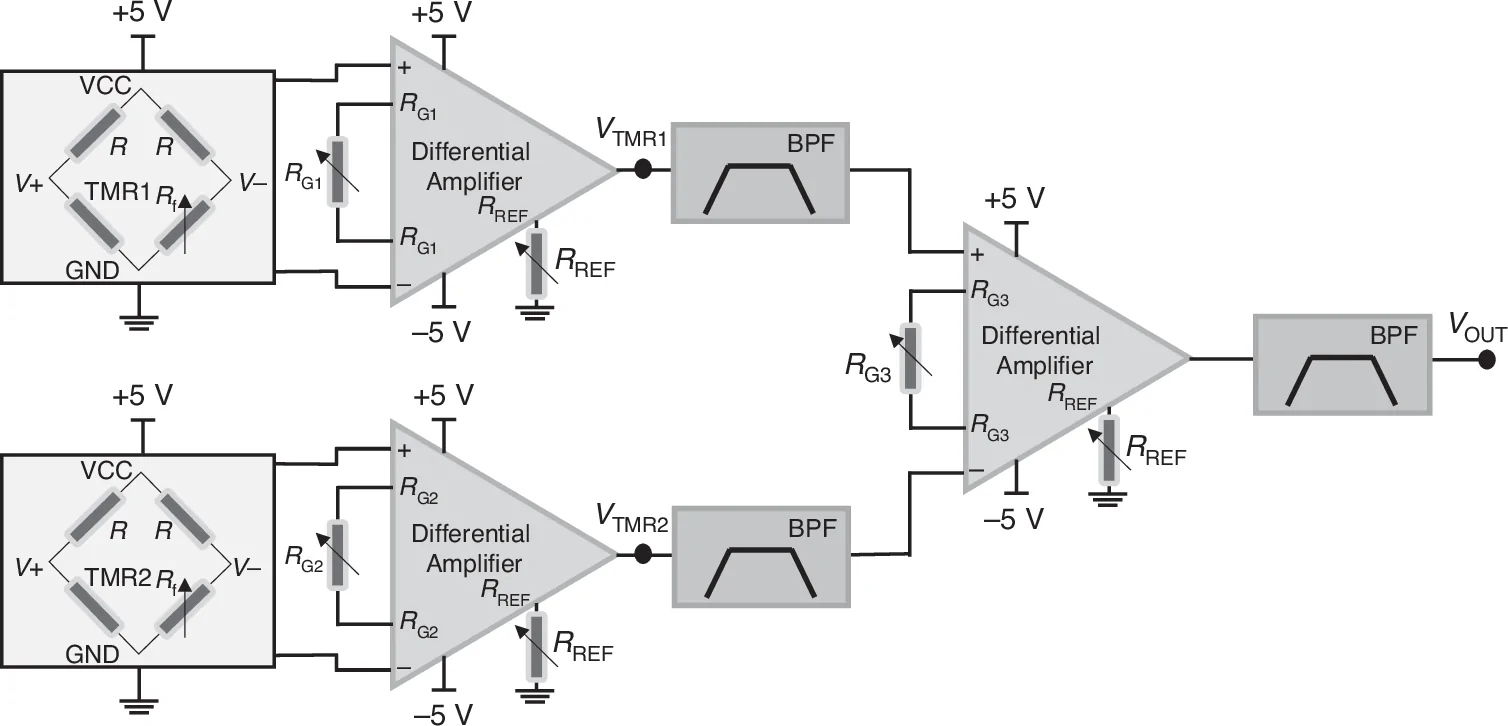
Schematic diagram of the low-noise differential interface circuit

To suppress environmental common-mode noise and compensate for device-to-device variations arising from micro- and nanofabrication processes (e.g., etching non-uniformity or metal redeposition), a two-stage differential interface circuit with dynamic sensitivity matching was designed. The sensing TMR and reference TMR were connected to two independent Wheatstone bridges, respectively, where sliding potentiometers were used to adjust the DC balance of each bridge to eliminate resistance mismatch. The bridge outputs were fed into the first-stage instrumentation amplifiers, in which the feedback gain ${{{\rm{G}}}}_{{{\rm{Amplifier}}}}$ was independently tuned to compensate for the intrinsic sensitivity differences between the two TMR elements. This configuration allowed the responses of the two channels to a uniform background magnetic field $({{{\rm{S}}}}_{{{\rm{TMR}}}}\times {{{\rm{G}}}}_{{{\rm{Amplifier}}}})$ to be matched as closely as possible, thereby improving the common-mode rejection ratio (CMRR). Subsequently, the amplified signals were processed by a 1–5 kHz band-pass filter, followed by a second-stage amplifier performing differential subtraction. The final output signal was further filtered and amplified using a low-noise SR560 preamplifier and digitized at a sampling rate of 30 kHz using a data acquisition card (Fig 13).

### Simulation-based evaluation of the effect of reference TMR spatial distribution on near-field magnetic signal detection

To quantitatively analyze the influence of the spatial distribution of the reference TMR element on near-field magnetic signal detection performance, a simulation experiment based on a microscale current source was conducted. A copper wire with a diameter of 30 μm was precisely positioned and fixed directly above the sensing region at the probe tip, serving as a near-field current source surrogate. A sinusoidal alternating current at 40 Hz was driven through the wire to generate a localized magnetic field exhibiting a steep spatial decay with distance. This excitation frequency was selected to provide a highly stable and reproducible test condition for geometric characterization. While maintaining constant source–sensor geometry and excitation amplitude, the responses of differential magnetrodes with different axial baseline lengths were systematically evaluated. By analyzing the signal amplitude attenuation and the corresponding signal-to-noise ratio (SNR) gain across different configurations, the impact of the reference TMR position on the near-field magnetic signal detection capability of the differential magnetrode was quantitatively assessed.

### Surgical and testing procedures

**Fig. 14:**
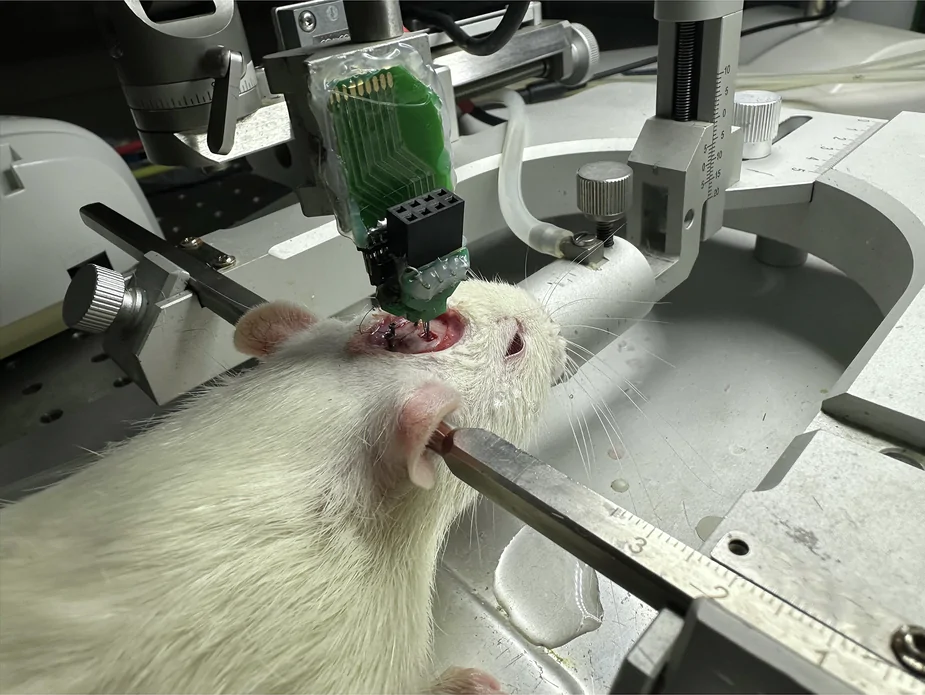
Surgical implantation of magnetrode into the rat hippocampus

Following the standard animal surgical procedures^37^, we performed the following steps. Male Sprague–Dawley (SD) rats weighing 250–300 g were used in this study. Animals were initially anesthetized with 5% isoflurane and fixed in a stereotaxic frame, with anesthesia maintained throughout the surgical procedure. After removal of the scalp and subcutaneous tissue, the skull surface was cleaned using 3% hydrogen peroxide to expose the cranial sutures. Stereotaxic coordinates were determined according to the rat brain atlas targeting the hippocampal CA1 region (AP = −2.76 mm, ML = +2.0 mm, DV = −2.76 mm relative to Bregma). A craniotomy (2 mm ×  1.5 mm) was performed at the target site using a dental drill. After removing the periosteum and incising the dura mater, the TMR-based magnetrode was precisely advanced to the target depth using a motorized microelectrode manipulator. To enhance recording stability and suppress environmental electromagnetic interference, four stainless steel skull screws were fixed to the skull and connected via copper wires to serve as a common ground. The implantation site was then sealed with dental cement (Fig 14). After a 24 h postoperative recovery period, the animals were placed inside an electromagnetic shielding cage for in vivo neural activity recording. To minimize motion artifacts and ensure signal stability, recordings were conducted under low-concentration isoflurane anesthesia. All animal experiments were carried out with the permission of the Beijing Association on Laboratory Animal Care (Beijing China) and approved by Institutional Animal Care and Use Committee at the Aerospace Information Research Institute, Chinese Academy of Sciences (AIRCAS, Beijing, China).

### In vitro cytotoxicity evaluation

HT22 mouse hippocampal neuronal cells were cultured in Dulbecco’s Modified Eagle Medium (DMEM) supplemented with 10% fetal bovine serum and 1% penicillin–streptomycin at 37 °C in a humidified atmosphere containing 5% CO₂. The culture medium was refreshed every 2–3 days, and cells were passaged upon reaching ~90% confluence.

For cytotoxicity assessment, sterilized samples were placed at the bottom of 48-well culture plates. Cells were seeded at a density of 1  × 10⁵ cells mL⁻¹ (300 μL per well) and incubated for 24 h. Three experimental groups were evaluated: (i) blank control without material, (ii) control material without passivation, and (iii) passivated experimental material. Cell viability was quantified using a CCK-8 assay. After incubation, the culture medium was replaced with 300 μL of CCK-8 working solution and incubated for 1 h at 37 °C. Subsequently, 100 μL of supernatant from each well was transferred to a 96-well plate, and absorbance was measured at 450 nm using a microplate reader.

### Live/dead cell staining

Live/dead staining was performed to visualize cell viability and morphology. HT22 cells were cultured in six-well plates under the same conditions described above. After removing the culture medium, cells were gently washed two to three times with 1× Assay Buffer to eliminate residual esterase activity. A staining solution containing Calcein-AM (2 μM) and propidium iodide (PI, 4.5 μM) was freshly prepared and added to each well. Cells were incubated at 37  °C for 15 min, washed with Assay Buffer, and imaged using an inverted fluorescence microscope. Live cells exhibited green fluorescence, whereas dead cells showed red fluorescence.

### Material implantation and histological analysis

For in vivo biocompatibility evaluation, sterilized samples were surgically implanted into the target brain regions of experimental animals. After the designated implantation period, animals were euthanized, and the implanted materials together with surrounding tissues were harvested and fixed. Samples were dehydrated through a graded ethanol series, cleared in xylene, and embedded in paraffin. Paraffin blocks were sectioned into 4-μm thick slices using a rotary microtome.

For hematoxylin and eosin (H&E) staining, tissue sections were deparaffinized, rehydrated, stained with hematoxylin for 1 min, rinsed and blued in running tap water, followed by eosin staining for 1 min. After dehydration and mounting with neutral resin, panoramic bright-field images were acquired using a digital imaging system to assess tissue morphology and inflammatory response around the implanted materials.

## Data Availability

All data needed to evaluate the conclusions in the paper are present in the paper and/or the Supplementary Materials. Additional data related to this paper may be requested from the authors.

## References

[1] Chamberland, S., Timofeeva, Y., Evstratova, A., Volynski, K. & Tóth, K. Action potential counting at giant mossy fiber terminals gates information transfer in the hippocampus. *Proc. Natl. Acad. Sci. USA* **115**, 7434–7439 (2018). 29946034 10.1073/pnas.1720659115 PMC6048548

[2] Yin, S. et al. Engineering 2D silicene-based mesoporous nanomedicine for in vivo near-infrared-triggered analgesia. *Adv. Sci.* **9**, 2202735 (2022). 10.1002/advs.202202735 PMC9443434 35750652

[3] Jiang, S. et al. Spatially expandable fiber-based probes as a multifunctional deep brain interface. *Nat. Commun.* **11**, 6115 (2020). 33257708 10.1038/s41467-020-19946-9 PMC7704647

[4] Scanziani, M. & Häusser, M. Electrophysiology in the age of light. *Nature* **461**, 930–939 (2009). 19829373 10.1038/nature08540

[5] Hong, G. & Lieber, C. M. Novel electrode technologies for neural recordings. *Nat. Rev. Neurosci.* **20**, 330–345 (2019). 30833706 10.1038/s41583-019-0140-6 PMC6531316

[6] Buzsáki, G., Anastassiou, C. A. & Koch, C. The origin of extracellular fields and currents — EEG, ECoG, LFP and spikes. *Nat. Rev. Neurosci.* **13**, 407–420 (2012). 22595786 10.1038/nrn3241 PMC4907333

[7] Einevoll, G. T., Kayser, C., Logothetis, N. K. & Panzeri, S. Modelling and analysis of local field potentials for studying the function of cortical circuits. *Nat. Rev. Neurosci.* **14**, 770–785 (2013). 24135696 10.1038/nrn3599

[8] Polikov, V. S., Tresco, P. A. & Reichert, W. M. Response of brain tissue to chronically implanted neural electrodes. *J. Neurosci. Methods* **148**, 1–18 (2005). 16198003 10.1016/j.jneumeth.2005.08.015

[9] Kozai, T. D. Y., Jaquins-Gerstl, A. S., Vazquez, A. L., Michael, A. C. & Cui, X. T. Brain tissue responses to neural implants impact signal sensitivity and intervention strategies. *ACS Chem. Neurosci.* **6**, 48–67 (2015). 25546652 10.1021/cn500256e PMC4304489

[10] Hämäläinen, M., Hari, R., Ilmoniemi, R. J., Knuutila, J. & Lounasmaa, O. V. Magnetoencephalography-theory, instrumentation, and applications to noninvasive studies of the working human brain. *Rev. Mod. Phys.* **65**, 413–497 (1993).

[11] Barry, J. F. et al. Optical magnetic detection of single-neuron action potentials using quantum defects in diamond. *Proc. Natl. Acad. Sci. USA* **113**, 14133–14138 (2016). 27911765 10.1073/pnas.1601513113 PMC5150388

[12] Baillet, S. Magnetoencephalography for brain electrophysiology and imaging. *Nat. Neurosci.* **20**, 327–339 (2017). 28230841 10.1038/nn.4504

[13] Brookes, M. J. et al. Magnetoencephalography with optically pumped magnetometers (OPM-MEG): the next generation of functional neuroimaging. *Trends Neurosci.* **45**, 621–634 (2022). 35779970 10.1016/j.tins.2022.05.008 PMC10465236

[14] Fred, A. L. et al. A brief introduction to magnetoencephalography (MEG) and its clinical applications. *Brain Sci.* **12**, 788 (2022). 35741673 10.3390/brainsci12060788 PMC9221302

[15] Hari, R. & Salmelin, R. Magnetoencephalography: from SQUIDs to neuroscience. *Neuroimage* **61**, 386–396 (2012). 22166794 10.1016/j.neuroimage.2011.11.074

[16] Clarke, J., Lee, Y.-H. & Schneiderman, J. Focus on SQUIDs in biomagnetism. *Supercond. Sci. Technol.* **31**, 080201 (2018).

[17] Boto, E. et al. Moving magnetoencephalography towards real-world applications with a wearable system. *Nature* **555**, 657–661 (2018). 29562238 10.1038/nature26147 PMC6063354

[18] Tierney, T. M. et al. Optically pumped magnetometers: From quantum origins to multi-channel magnetoencephalography. *Neuroimage* **199**, 598–608 (2019). 31141737 10.1016/j.neuroimage.2019.05.063 PMC6988110

[19] Christopher, G. et al. Magnetoencephalography with optically pumped magnetometers (OPM-MEG): studying brain activity in neurological patients while standing. *J. Neurol. Neurosurg. Psychiatry* **95**, A64–A64 (2024).

[20] Su, D., Wu, K., Saha, R., Peng, C. & Wang, J.-P. Advances in magnetoresistive biosensors. *Micromachines.* **11**, 34 (2019). 31888076 10.3390/mi11010034 PMC7019276

[21] Lenz, J. & Edelstein, S. Magnetic sensors and their applications. *IEEE Sens. J.* **6**, 631–649 (2006).

[22] Barbieri, F. et al. Local recording of biological magnetic fields using giant magneto resistance-based micro-probes. *Sci. Rep.* **6**, 39330 (2016). 27991562 10.1038/srep39330 PMC5171880

[23] Valadeiro, J. et al. Microneedles with integrated magnetoresistive sensors: a precision tool in biomedical instrumentation. In *2017 IEEE Sensors Applications Symposium (SAS)* 1–6 (IEEE, 2017).

[24] Caruso, L. et al. In vivo magnetic recording of neuronal activity. *Neuron* **95**, 1283–1291.e4 (2017). 28844526 10.1016/j.neuron.2017.08.012 PMC5744593

[25] Chopin, C. et al. Magnetoresistive sensor in two-dimension on a 25 μm thick silicon substrate for in vivo neuronal measurements. *ACS Sens.* **5**, 3493–3500 (2020). 33108725 10.1021/acssensors.0c01578

[26] Klein, F. J. et al. In vivo magnetic recording of single-neuron action potentials. *J. Neurophysiol.* **134**, 1306–1319 (2025). 40953071 10.1152/jn.00491.2024

[27] Luo, J. et al. Development and comprehensive evaluation of TMR sensor-based magnetrodes. *ACS Appl. Mater. Interfaces* **16**, 31677–31686 (2024). 38833518 10.1021/acsami.4c01148

[28] Roth, B. J. & Wikswo, J. P. The magnetic field of a single axon. A comparison of theory and experiment. *Biophys. J.* **48**, 93–109 (1985). 4016213 10.1016/S0006-3495(85)83763-2 PMC1329380

[29] Lv, S. et al. Tentacle microelectrode arrays uncover soft boundary neurons in hippocampal CA1. *Adv. Sci.* **11**, 2401670 (2024). 10.1002/advs.202401670 PMC11304256 38828784

[30] Wang, Y. et al. Enhanced neural activity detection with microelectrode arrays modified by drug-loaded calcium alginate/chitosan hydrogel. *Biosens. Bioelectron.* **267**, 116837 (2025). 39369514 10.1016/j.bios.2024.116837

[31] Soltesz, I. & Losonczy, A. CA1 pyramidal cell diversity enabling parallel information processing in the hippocampus. *Nat. Neurosci.* **21**, 484–493 (2018). 29593317 10.1038/s41593-018-0118-0 PMC5909691

[32] Hill, D. N., Mehta, S. B. & Kleinfeld, D. Quality metrics to accompany spike sorting of extracellular signals. *J. Neurosci.* **31**, 8699–8705 (2011). 21677152 10.1523/JNEUROSCI.0971-11.2011 PMC3123734

[33] Pachitariu, M., Sridhar, S., Pennington, J. & Stringer, C. Spike sorting with Kilosort4. *Nat. Methods* **21**, 914–921 (2024). 38589517 10.1038/s41592-024-02232-7 PMC11093732

[34] Henze, D. A. et al. Intracellular features predicted by extracellular recordings in the hippocampus in vivo. *J. Neurophysiol.* **84**, 390–400 (2000). 10899213 10.1152/jn.2000.84.1.390

[35] Bean, B. P. The action potential in mammalian central neurons. *Nat. Rev. Neurosci.* **8**, 451–465 (2007). 17514198 10.1038/nrn2148

[36] Zhang, J., Luo, J., Zou, X. & Chen, J. An endpoint detection system for ion beam etching using optical emission spectroscopy. *Micromachines* **13**, 259 (2022). 35208383 10.3390/mi13020259 PMC8877325

[37] Jing, L. et al. Deep brain implantable microelectrode arrays for detection and functional localization of the subthalamic nucleus in rats with Parkinson’s disease. *Bio-Des. Manuf.* **7**, 439–452 (2024).

